# Psychosocial consequences among women with false-positive results after mammography screening in Norway

**DOI:** 10.1080/02813432.2018.1523985

**Published:** 2018-10-08

**Authors:** Marit Solbjør, Siri Forsmo, John-Arne Skolbekken, Volkert Siersma, John Brodersen

**Affiliations:** a Department of Public Health and Nursing, Trondheim, Norway;; b The Research Unit for General Practice and Section of General Practice, Department of Public Health, University of Copenhagen, Copenhagen, Denmark;; c Primary Health Care Research Unit, Region Zealand, Denmark

**Keywords:** Mammography, screening, false-positive, psycho-social consequences

## Abstract

**Background:** Mammography screening may cause psychosocial harm for women experiencing a false-positive screening result. Previous studies suggest long-term consequences. The aim of the present study was to assess psychosocial consequences of false-positive findings on screening mammography within a six month follow-up.

**Methods:** A prospective matched cohort survey study using the questionnaire ‘Consequences of Screening for Breast Cancer’ (COS-BC), which was translated from Danish to Norwegian. Psychometric analyses investigated the measurement properties of the Norwegian version. Two screening clinics in Norway distributed the survey to 299 women with an abnormal mammogram and 541 women with a normal screen. Women received the questionnaire when receiving the screening result, and one and six months after screening.

**Results:** At six months, statistically significant differences appeared in two scales: *existential values* and *breast examination*. At six-month follow-up, women with false-positive results showed no statistically significant differences from women diagnosed with breast cancer in three outcomes: *sense of dejection, anxiety*, and *keeping my mind off things*.

**Conclusion:** Our results indicate that the psychosocial consequences from having false-positive screening mammography results diminish after six months. The results support previous research describing breast-specific outcomes. However, our results indicate that Norwegian women are less frightened than other Scandinavian mammography screening participants.

## Introduction

In cancer screening, some individuals who do not have cancer are subject to follow-up examinations due to an abnormal screening result. Having a false-positive screening mammography (hereafter referred to as false positives) may lead to adverse psychosocial consequences. Several reviews have concluded that short-term adverse psychological consequences of mammography screening are significantly higher for women with false positives than for women who receive normal screening results in the first three months after mammography [1,2]. Long-term consequences are contested. Several studies have found no increased long-term levels of generalized anxiety among women who experience false positives [3–10]. Other studies report that anxiety was greater among false positives compared to women with normal results in a 12 months period [7,11], or even up to 24 months [12]. One review concluded that there were no long-term symptoms of generalized depression among women with false positives [13]. A Norwegian study among women who experienced a recall found transiently increased anxiety and a slight increase in depression. Four weeks after screening the level of anxiety was the same as that of the general female Norwegian population, whereas depression levels were lower [14]. A more recent Norwegian study found no effect on anxiety but increased depression as long as six months after false positive diagnoses [15]. In a Danish longitudinal study, women with false positives reported changes in existential values and inner calmness as great as those reported by women diagnosed with breast cancer at six-month follow-up [16]. Three years after being declared cancer free, women with false positives consistently reported greater negative psychosocial consequences than women who had normal findings [16].

Variance in psychosocial consequences from screening in different studies could be caused by what is measured, and how [17]. There has been a need for studies using adequate tools for measuring psychosocial consequences from having a false-positive result from screening, as such tools must cover anxiety, fear, mood, behaviour, sleep, sexuality and social functioning [18]. While studies using generic measures designed to measure general anxiety and depression at clinically relevant levels do not find significant negative psychological impact from having false positives, those using condition-specific instruments do [2]. In a meta-analysis, having false positives had little effect on generalized outcomes, but had significant effects on breast-cancer specific outcomes, including distress about breast cancer, somatization or symptoms in the breast, fear of getting breast cancer, anxiety and worry about breast cancer, perceived benefits of mammography, and frequency of breast self-exams [1]. To meet the need for a condition-specific instrument for mammography screening, the questionnaire ‘Consequences of screening’ [4] was developed and validated into ‘Consequences of screening – breast cancer (COS-BC)’ to fit studies on breast cancer [19]. Previous international research has shown that having a false-positive result after mammography screening can have short-term, breast specific psychosocial consequences. In this study we present results from the Norwegian version of the COS-BC to examine the psychosocial consequences of a false-positive result in a Norwegian population.

## Aim of study

The overall aim of this study was to assess psychosocial consequences of false-positive findings on screening mammography within a six-month follow-up. To do so adequately, the Danish version of COS-BC was translated and adapted into Norwegian, and the domains were tested for reliability and unidimensionality using Rasch modeling.

## Methods and material

### Study context and population

The Norwegian Breast Cancer Screening Programme invites all women aged 50–69 years to biennial mammography. Between 1996 and 2010, more than two million screening examinations were performed in the programme [20]. Participation is covered by the national health insurance, with the exception of an individual payment of approximately 200 Norwegian kroner. Within the screening programme, the cumulative risk of false positives has been found to be 23%, with the rate for false positive screening results varying between 1.8% and 4.1% across screening units [20].

### The questionnaire and translation to Norwegian

COS-BC consists of two parts. Part-I encompasses 29 items: two single items (‘Felt less attractive’ and ‘Busy to take my mind off things’) and six scales measuring anxiety (6 items), sense of dejection (6 items), negative impact on behaviour (7 items), sleep (4 items), degree of breast self-examination (2 items), and sexuality (2 items) [21]. All items in Part-I have four response categories: ‘not at all’, ‘a bit’, ‘quite a bit’ and ‘a lot’. The higher the score of the outcome, the more negative psychosocial consequences the person has experienced [21]. Part-II consists of 13 items for those who had a screening negative or a false-positive result, while two items concerning belief in having breast cancer were left out for those diagnosed with breast cancer. Part-II encompasses four scales: existential values (6 items), relationships within social network (3 items), being less or more relaxed/calm (2 items), and being less or more anxious about breast cancer/belief in having or not-having breast cancer (2 items) [22]. All items in these scales have five response categories: ‘Much less’, ‘Less’, ‘The same as before’, ‘More’, and ‘Much more’. Changes following existential crisis can be interpreted by the individual as positive or negative, or a combination of both. Therefore, Part-II requires a ‘laterally reversed’ scoring system: A response to ’The same as before’ becomes a value of ‘No change’, a response to ‘Less’ or ‘More’ becomes a value of ‘Minor change’, and a response to ‘Much less’ or ‘Much more’ becomes a value of ‘Major change’ [22,23]. The four sum-scores of Part-II reflect the degree of changes in the four long-term psychosocial outcomes respectively. A high score in part-II denotes that the individual is highly psychosocially affected, irrespective of this being a positive or negative experience [22,23].

The Danish COS-BC was the original version of the questionnaire [22]. It has demonstrated adequate psychometric measurement properties: high content validity, evidence of uni-dimensionality, and limited DIF founding in the Rasch analysis. It was translated and adapted into Norwegian using the dual panel method [24]. The original version was translated into Norwegian in a consensus meeting among three bilingual people moderated by JB. If an item could be translated into Norwegian in two or more ways, they were allowed to suggest these alternatives to the next panel. Second, this Norwegian draft version of the COS-BC was tested in a lay panel. Criteria for the five lay panel members were to have Norwegian as their first language, no particular knowledge of Danish, no health-related education, no higher education, and being eligible for the mammography programme. Any changes suggested by the lay panel were incorporated. If the bilingual panel had suggested two or more translations, the lay panel decided which was closest to Norwegian lay language. Finally, this second draft version of the Norwegian COS-BC was tested in single interviews with ten women who had participated in the mammography programme. Three interviewees had previously experienced false positives. While completing the questionnaire, they discussed it with a researcher, using the ‘think-aloud’ method [25]. All found the questionnaire easy to complete. The heading ‘to women who have been invited to mammography’ was changed to ‘to women who have been to mammography’. Three women found it hard to answer the question ‘time has felt long’, which was changed to ‘time has passed slowly’. No other changes were made to the questionnaire. The Norwegian version of COS-BC do not show variance in the Rasch analyses compared to the Danish version.

### Study design and survey administration

The Regional Committee for Medical and Health Research Ethics approved the study. The survey was administered through two regional hospitals in North and Central Norway, which provides all mammography screening recall examinations in their region. All women who had an abnormal screening mammography during the study period April through December 2010 received the questionnaire. For each recalled woman included, two women with negative findings who had been screened at the same date and unit were additionally included in the study. Due to summer closing, 29 screen positives were not matched with screen negative women.

The two hospitals had different handling time for interpreting mammographic images, which led to two sampling procedures. At one hospital, women received a letter including their screening result one week after the mammography examination. Screen positives received their first questionnaire together with their recall letter. Screen negatives received their first questionnaire two weeks after the letter with screening results had been sent. At the other hospital, women had their screening result within four weeks after the examination. Screen positive women received their first questionnaire together with their recall letter, whereas the control group received their first questionnaire 4–5 weeks after screening.

**Figure 1.**
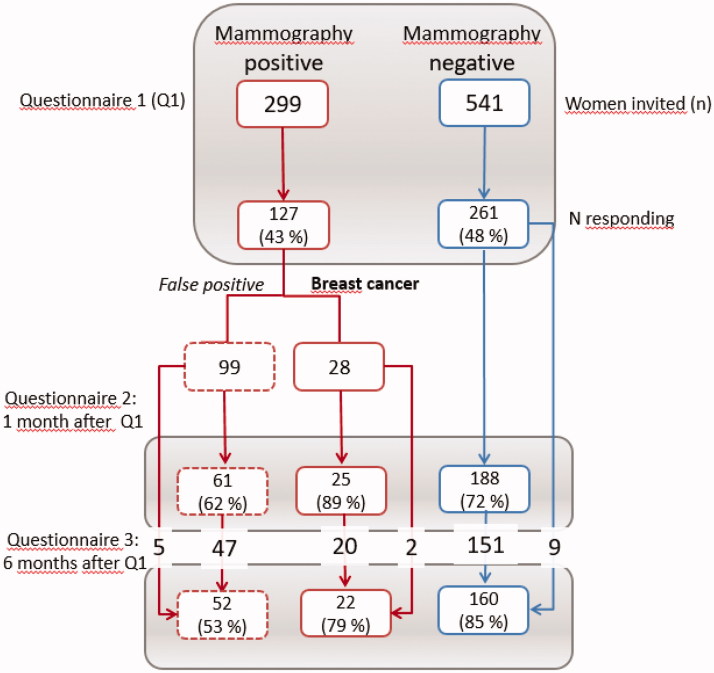
Flow-chart.

All questionnaires were sent from the hospital to the women’s home addresses and returned in a prepaid envelope. Returning the first questionnaire was seen as giving informed consent to participate in the study. Women who returned their first questionnaire received the COS-BC at two additional follow-up time points: One and six months after receiving their screening results. Only women who returned the second questionnaire received the third. Through an inadvertence, some women received questionnaire 3 without having answered questionnaire 2 (see Figure 1). A secretary at each hospital assigned numbers to each woman and kept track of their diagnostic status. Researchers did not know the identity of the women and the hospital staff could not see the responses.

Three sociodemographic variables were registered: the region where the women lived signified by the hospital the women had been examined at; the degree of urbanization in which the women lived signified by their postal code; and their age.

## Statistical methods

### Psychometric analysis

The psychometric properties of the 10 COS-BC scales were tested for homogeneity and differential item functioning (DIF) relative to participant age by using likelihood ratio tests on appropriately conditioned Rasch models at the 1 month follow-up time point [1]. Reliability of the scales was examined using Cronbach’s alpha [26]. The software programme DIGRAM was used for these psychometric analyses [27].

### Longitudinal analysis

A COS-BC dimension was set to missing for a certain time point in the follow-up if one or more items comprising the dimension were missing at the corresponding time point. Also the response category ‘not applicable’ in the sexuality dimension counted as missing in this respect.

Multivariable linear regression was used to analyse the differences in the developments of each of the COS-BC dimensions over the three follow-up time points between the screening groups; the analyses were adjusted for the baseline factors. To adjust for a possible different response pattern in the three groups, the non-missing dimensions at each time point were weighted by the inverse of an estimate of the probability of this score being observed. These probabilities were estimated in logistic regression models for the domain being missing, with the baseline covariates, the screening groups, and the observed scores of the corresponding domain at previous time points as covariates. Generalized estimating equations were used to adequately adjust the covariance for repeated measurement and weighting.

SAS 9.4 was used for the longitudinal analyses with a statistical significance level of *p* < 0.01. Three comparisons were done in the analysis: between women with negative and false-positive findings, between women with negative screening results and women with breast cancer, and between women with false positives and women with breast cancer. Each comparison was done at the three assessments points.

## Results

COS-BC was sent to 299 women who had an abnormal screening mammography, of which 127 women participated in the first round (99 with false positives and 28 women with breast cancer). At one-month follow-up 61 women with false positives and 25 women with breast cancer completed the COS-BC, and 52 and 22 women participated at six months, respectively. As controls, 541 women with normal screening results received the first questionnaire, of which 261 were returned, 188 at one month, and 160 after six months. We found no statistically significant differences between the three screening groups in relation to age, urban/rural residence, or to which hospital they belonged (Table 1).

**Table 1.** Baseline characteristics of the screened population.

Screening result						
		Total	Normal	False positive	Breast cancer	
		(*n* = 388)	(*n* = 261)	(*n* = 99)	(*n* = 28)	
	*n/n/n*	*n* (%)	*n* (%)	*n* (%)	*n* (%)	*p*-Value
Age						
51–55	261/99/28	125 (32.2)	82 (31.4)	39 (39.4)	4 (14.3)	0.3420
56–60		92 (23.7)	62 (23.8)	22 (22.2)	8 (28.6)	
61–65		103 (26.6)	71 (27.2)	22 (22.2)	10 (35.7)	
66–70		68 (17.5)	46 (17.6)	16 (16.2)	6 (21.4)	
Residence						
Rural	261/99/28	156 (40.2)	98 (37.6)	47 (47.5)	11 (39.3)	0.2285
Urban		232 (59.8)	163 (62.5)	52 (52.5)	17 (60.7)	
Hospital						
Bodø	261/99/28	224 (57.7)	155 (59.4)	52 (52.5)	17 (60.7)	0.4736
Trondheim		164 (42.3)	106 (40.6)	47 (47.5)	11 (39.3)	

The 10 Norwegian COS-BC scales exhibited overall fit to the partial credit Rasch model for polytomous items (Table 2). No DIF was revealed and Cronbach’s Alpha was 0.652–0.926 (Table 2).

**Table 2.** Conditional likelihood ratio (CLR) fit statistics and Cronbach's alpha for the 10 domains of the Norwegian Consequences of Screening – Breast Cancer (COS-BC) questionnaire.

	Scales (no. of items)	CLR	Degrees of freedom	p*	Cronbach's alpha
1	Anxiety [6]	12.8	15	0.617	0.921
2	Behaviour [7]	11.0	17	0.858	0.878
3	Dejection [6]	2.4	17	0.990	0.918
4	Negative impact on sleep [4]	11.3	11	0.418	0.926
5	Breast self-examination [2]	0.8	5	0.977	0.789
6	Sexuality [2]	10.3	5	0.068	0.813
7	Less or more anxious about breast cancer [2]	1.1	3	0.782	0.652
8	Calm/relaxed [2]	1.3	3	0.731	0.700
9	Social network [3]	2.0	5	0.852	0.672
10	Existential values [6]	23.3	11	0.016	0.925

*After adjustment for multiple testing by using the methods of Benjamini–Hochberg, the level of statistical significance was assessed at a level of 0.05.

### Normal screening results compared to breast cancer diagnosis

The differences in psychosocial consequences between women who had a normal screening result and women diagnosed with breast cancer persisted throughout one and six months (Figure 2, Table 3).

Figure 2.The mean score of each of the 8 psychosocial outcomes, part I of the COS-BC for the 3 screening groups at 3 time points: 0, 1, and 6 months.

**Figure:**
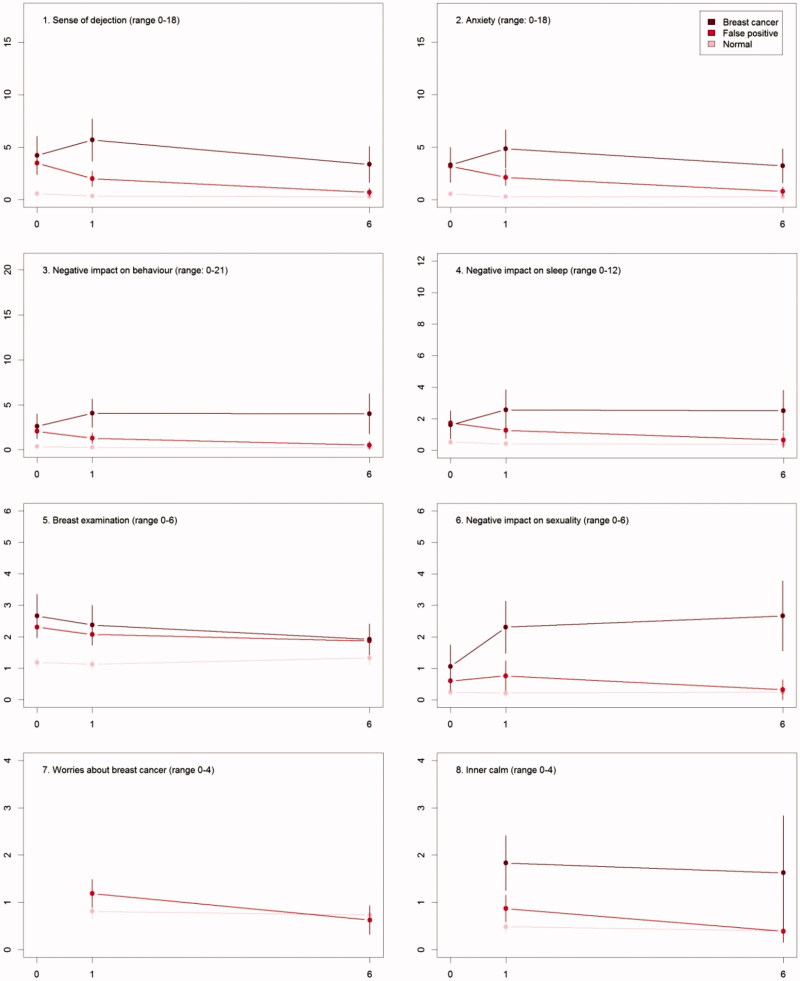

**Figure:**
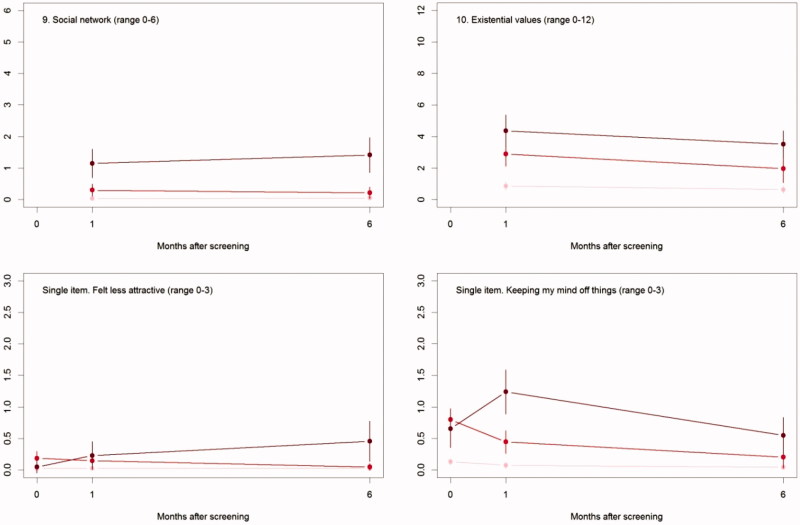

**Table 3.** Adjusted analyses of psycho-social consequences of breast cancer screening: estimated differences between each pair of the three diagnosis groups.

		Follow-up time					
		0 months		1 month		6 months	
Scale (range)	Difference	Mean (95% CI)	*p*-Value	Mean (95% CI)	*p*-Value	Mean (95% CI)	*p*-Value
1: Sense of dejection (0–18)	Normal → Breast cancer	3.73 (1.94; 5.53)	0.0000	5.46 (3.45; 7.48)	0.0000	3.11 (1.43; 4.80)	0.0003
	Normal → False positive	2.92 (2.19; 3.65)	0.0000	1.68 (0.91; 2.46)	0.0000	0.44 (0.02; 0.85)	0.0407
	False positive → Breast cancer	0.81 (−1.10; 2.72)	0.4072	3.78 (1.62; 5.94)	0.0006	2.68 (0.96; 4.39)	0.0022
	*n*	*384*		*264*		*235*	
2: Anxiety (0–18)	Normal → Breast cancer	2.84 (1.17; 4.50)	0.0008	4.66 (2.85; 6.47)	0.0000	3.00 (1.39; 4.61)	0.0003
	Normal → False positive	2.67 (1.94; 3.41)	0.0000	1.86 (1.04; 2.68)	0.0000	0.52 (0.08; 0.97)	0.0215
	False positive → Breast cancer	0.17 (−1.63; 1.96)	0.8562	2.80 (0.82; 4.77)	0.0055	2.47 (0.83; 4.12)	0.0031
	*n*	*385*		*265*		*235*	
3: Negative impact on behaviour (0–21)	Normal → Breast cancer	2.36 (1.00; 3.73)	0.0007	3.88 (2.28; 5.47)	0.0000	3.78 (1.59; 5.97)	0.0007
	Normal → False positive	1.69 (1.07; 2.30)	0.0000	0.97 (0.32; 1.62)	0.0036	0.24 (−0.25; 0.72)	0.3404
	False positive → Breast cancer	0.68 (−0.80; 2.16)	0.3699	2.91 (1.20; 4.63)	0.0009	3.54 (1.33; 5.75)	0.0017
	*n*	*382*		*263*		*235*	
4: Negative impact on sleep (0–12)	Normal → Breast cancer	1.07 (0.16; 1.99)	0.0213	2.12 (0.83; 3.41)	0.0013	2.08 (0.68; 3.48)	0.0035
	Normal → False positive	1.22 (0.73; 1.71)	0.0000	0.86 (0.32; 1.41)	0.0019	0.24 (−0.27; 0.75)	0.3613
	False positive → Breast cancer	−0.15 (−1.15; 0.85)	0.7710	1.26 (−0.13; 2.65)	0.0747	1.84 (0.38; 3.31)	0.0136
	*n*	*385*		*265*		*235*	
5: Breast examination (0–6)	Normal → Breast cancer	1.47 (0.76; 2.19)	0.0000	1.26 (0.60; 1.91)	0.0002	0.55 (−0.00; 1.10)	0.0516
	Normal → False positive	1.13 (0.78; 1.49)	0.0000	0.98 (0.64; 1.33)	0.0000	0.58 (0.18; 0.98)	0.0041
	False positive → Breast cancer	0.34 (−0.43; 1.11)	0.3866	0.27 (−0.44; 0.99)	0.4504	−0.03 (−0.66; 0.59)	0.9159
	*n*	*386*		*267*		*235*	
6: Negative impact on sexuality (0–6)	Normal → Breast cancer	0.83 (0.16; 1.50)	0.0153	2.11 (1.29; 2.93)	0.0000	2.31 (1.32; 3.30)	0.0000
	Normal → False positive	0.39 (0.06; 0.71)	0.0191	0.55 (0.09; 1.02)	0.0197	0.03 (−0.33; 0.38)	0.8776
	False positive → Breast cancer	0.44 (−0.28; 1.16)	0.2277	1.56 (0.64; 2.48)	0.0009	2.28 (1.25; 3.31)	0.0000
	*n*	*324*		*231*		*209*	
Felt less attractive (0–3)	Normal → Breast cancer	0.03 (−0.07; 0.13)	0.5699	0.20 (−0.02; 0.43)	0.0782	0.45 (0.13; 0.76)	0.0051
	Normal → False positive	0.18 (0.07; 0.29)	0.0018	0.13 (0.01; 0.26)	0.0415	0.03 (−0.04; 0.09)	0.4206
	False positive → Breast cancer	−0.15 (−0.29; 0.00)	0.0445	0.07 (−0.19; 0.33)	0.6073	0.42 (0.10; 0.74)	0.0093
	*n*	385		271		235	
Keeping my mind off things (0–3)	Normal → Breast cancer	0.53 (0.23; 0.84)	0.0005	1.19 (0.85; 1.53)	0.0000	0.49 (0.20; 0.78)	0.0009
	Normal → False positive	0.67 (0.49; 0.85)	0.0000	0.37 (0.18; 0.56)	0.0001	0.15 (−0.02; 0.32)	0.0805
	False positive → Breast cancer	−0.14 (−0.48; 0.21)	0.4355	0.82 (0.44; 1.20)	0.0000	0.34 (0.01; 0.68)	0.0466
	*n*	385		273		235	
7: Worried about breast cancer (0–4)	Normal → Breast cancer	*		*		*	
	Normal → False positive	*		0.33 (−0.02; 0.67)	0.0615	−0.15 (−0.54; 0.23)	0.4319
	False positive → Breast cancer	*		*		*	
	*n*	0		255		209	
8: Inner calm (0–4)	Normal → Breast cancer	*		1.29 (0.76; 1.82)	0.0000	1.08 (0.12; 2.04)	0.0270
	Normal → False positive	*		0.39 (0.07; 0.71)	0.0171	−0.02 (−0.30; 0.26)	0.8790
	False positive → Breast cancer	*		0.90 (0.31; 1.49)	0.0028	1.10 (0.13; 2.07)	0.0256
	*n*	0		281		227	
9: Social network (0–6)	Normal → Breast cancer	*		1.10 (0.13; 2.07)	0.0000	1.32 (0.82; 1.82)	0.0000
	Normal → False positive	*		0.28 (0.08; 0.47)	0.0050	0.17 (−0.03; 0.36)	0.0949
	False positive → Breast cancer	*		0.82 (0.35; 1.29)	0.0006	1.16 (0.63; 1.68)	0.0000
	*n*	0		281		226	
10: Existential values (0–12)	Normal → Breast cancer	*		3.46 (2.41; 4.50)	0.0000	2.78 (1.85; 3.70)	0.0000
	Normal → False positive	*		2.09 (1.28; 2.90)	0.0000	1.30 (0.45; 2.16)	0.0028
	False positive → Breast cancer	*		1.37 (0.11; 2.63)	0.0337	1.47 (0.29; 2.66)	0.0149
	*n*	0		281		228	

### False positives compared to normal screening results

There were differences between women with a normal screen and false positives at baseline with respect to all scales, except *Negative impact on sexuality*. At six months, the only significant differences were for the two scales: *Breast examination* and *Existential values*. See Table 3.

### False positives compared to breast cancer diagnosis

At baseline, there were no differences between women who had false positives and diagnosed with breast cancer. From baseline to one month, differences increased on all scales and items. Between one and six months, differences increased between the two groups, except for two scales and one single item: *Sense of dejection*, *Anxiety*, and *Keeping my mind off things*, which all showed larger differences at one month than at six months.

## Discussion

The present study included a small population with a low response rate: 43% for screen positives and 48% for women with a normal screening result, which is a limitation. An indication of selection bias is the same age in all the sub-groups: it was expected that women diagnosed with breast cancer were older. We cannot adjust for this possible selection bias as we had no socio-demographic data on non-responders. Dropout rates indicate that the study was most relevant for women diagnosed with breast cancer. A potential lack of relevance for those with a normal screening result was supported by comments from women in the validation test panel who had not been recalled after mammography screening. In our study women with false positives had the lowest participation rate after six months. This is different from studies from the other Scandinavian countries [16,28]. After six months, the Danish study obtained a participation rate of 73.9%, the Swedish study 71%, while the present study only obtained a participation rate of 53% among women with false positives. Inviting women with a positive finding from mammography screening to answer questions about psychosocial consequences may have ethical implications. Some women might find such questions disturbing, reinforcing negative feelings. However, since mammography screening is targeting non-symptomatic women, the importance of exploring its consequences is vital in order to attend to women who are invited to screening.

Other limitations to the study are that there are few background variables and no registration of socio-economic status of study participants, and that 29 women with a positive screening result could not be matched with a comparative selection due to summer closing at one hospital. The latter implies a slight drop in power, but does not bias the results. All analyses were performed using the method of generalized estimating equations so as to adjust for the excess correlation of observations within matched groups. Data was collected in 2010. Since then, information leaflets from the Norwegian Breast Cancer Screening Programme have been redesigned. This may influence how women experience their screening participation. However, the organization of the mammography screening programme and the recall process is the same in 2018 as in 2010, which suggest that our results remain valid. Women with positive screening results got the questionnaire together with the recall letter and were encouraged in the information letter to complete the questionnaire before attending their recall examination. It is however impossible to know if some waited until after the examination. Women who had normal screening results received the COS-BC questionnaire two to five weeks after receiving their result from the examination. Variance in timing of completing the questionnaire could have influenced their answers, but previous research indicate that receiving a ‘no-findings’ message is unlikely to cause psychological consequences [29].

Previous research indicates that having false positives may lead to negative psychosocial consequences. A meta-analysis found that although there were minor psychosocial consequences identified via generalized measures, false positives had an influence on breast-specific items [1]. In the present study, women who experienced false positives differed from women with a normal screen on changes in two scales at 6-month follow-up: *Existential values* and *Breast examination* with a difference in score of 1.3 and 0.58 respectively. For example, a statistically significant mean increase of a score of 1 in the scale of Existential values corresponds to a shift in responses from ‘no changes’ to ‘minor changes’ or from ‘minor changes’ to ‘major changes’ in one item in this scale for every women having false positives. We think that such a change is of social and clinical significance. One explanation for this is that these women have been subject to a raised awareness of their personal breast cancer risk [30,31]. Another explanation is that women in an opt-out mammography screening programme have not considered their risk of a recall when entering the programme [13]. Women may participate in screening to confirm that they are free of breast cancer [30,32]. After six months, women with false positives did not differ from those with a normal screening result on the item ‘worried about breast cancer’. One explanation is the selection bias plus the low response rates. This is supported by Swedish and Danish studies, which found that those most positive to screening have higher participation rates than those who are more worried [16,28].

We found no statistically significant differences between women who had false positives and those who were diagnosed with breast cancer, regarding breast examination after six months. This indicates that false positives may influence awareness about breast cancer in healthy women. However, in the scale *Breast examination* there were no statistically significant differences between women with normal screening results and women with breast cancer. This could be due to a type 2-error because of the small number of participants with breast cancer, which is a limitation for all our findings comparing women with breast cancer to the two other groups.

Existential values remained significantly different between women with false positives and women with normal screening results at 6-month follow-up. This shows the ability of COS-BC to detect subtle changes from having false positives. Qualitative studies find that women’s experiences from false positives are varied and multifaceted [30–32]. These changes and experiences may be difficult to measure quantitatively. We cannot know if the change in existential values were experienced positively or negatively by the women. However, in our interpretation, changes among non-symptomatic individuals due to participation is a negative feature of screening.

The Danish and Swedish studies with COS-BC found significant differences between women with false positives and normal screening results in some of the scales in part-I of COS-BC [16,28]. Our study did not find the same level of negative psychosocial consequences. Other Norwegian studies indicate similar trends. One such study indicated that the anxiety level was the same and depression was lower for those with a false-positive screen compared to the general female Norwegian population, four weeks after screening [14]. Another study found no effect on anxiety, but increased depression six months after having false positives [15]. Bias in recruitment, challenges with the questionnaire, or the lack of possibility to adjust for potential confounding such as socio-demographics could explain why Norwegian women seem less frightened by having false positives than other Scandinavian women. Other explanations might be how information is provided before screening, or trust in health services. Because psychosocial effects have the potential to affect many of the women participating in mammography screening, it is important to make these questions the subject of further research.

## Conclusion

This study found psychosocial consequences for women who have false positives at one month, but only in two of twelve psychosocial outcomes at six months. Our study adds to previous research on indicating that false positives may lead to increased worries on breast cancer-specific items as long as six months after participating in mammography screening.

## Ethical approval

The study was approved by the Regional Committee for Medical and Health Research Ethics, registration number 4.2009.301.

## References

[1] Salz T, Richman AR, Brewer NT Meta-analyses of the effect of false-positive mammograms on generic and specific psychosocial outcomes. Psychooncology. 2010;19(10):1026–1034. 20882572 10.1002/pon.1676

[2] Bond M, Pavey T, Welch K, et al. Systematic review of the psychological consequences of false-positive screening mammograms. Health Technol Assess (Winchester, England). 2013;17(13):1–170. 10.3310/hta17130 PMC4781086 23540978

[3] Gram IT, Lund E, Slenker SE Quality of life following a false positive mammogram. Br J Cancer. 1990;62(6):1018–1022. 2257206 10.1038/bjc.1990.430 PMC1971570

[4] Cockburn J, Staples M, Hurley SF, et al. Psychological consequences of screening mammography. J Med Screen. 1994;1(1):7–12. 8790480 10.1177/096914139400100104

[5] Lowe JB, Balanda KP, Del Mar C, et al. Psychologic distress in women with abnormal findings in mass mammography screening. Cancer. 1999;85(5):1114–1118. 10091796 10.1002/(sici)1097-0142(19990301)85:5<1114::aid-cncr15>3.0.co;2-y

[6] Ekeberg O, Skjauff H, Karesen R Screening for breast cancer is associated with a low degree of psychological distress. Breast. 2001;10(1):20–24. 14965553 10.1054/brst.2000.0177

[7] Lampic C, Thurfjell E, Bergh J, et al. Short- and long-term anxiety and depression in women recalled after breast cancer screening. Eur J Cancer (Oxford, England: 1990). 2001;37(4):463–469. 10.1016/s0959-8049(00)00426-3 11267855

[8] Sandin B, Chorot P, Valiente RM, et al. Adverse psychological effects in women attending a second-stage breast cancer screening. J Psychosom Res. 2002;52(5):303–309. 12023127 10.1016/s0022-3999(01)00227-6

[9] Scaf-Klomp W, Sanderman R, van de Wiel HB, et al. Distressed or relieved? Psychological side effects of breast cancer screening in The Netherlands. J Epidemiol Community Health. 1997;51(6):705–710. 9519137 10.1136/jech.51.6.705 PMC1060571

[10] Aro AR, Pilvikki Absetz S, van Elderen TM, et al. False-positive findings in mammography screening induces short-term distress – breast cancer-specific concern prevails longer. Eur J Cancer (Oxford, England: 1990). 2000;36(9):1089–1097. 10.1016/s0959-8049(00)00065-4 10854941

[11] Hislop TG, Harris SR, Jackson J, et al. Satisfaction and anxiety for women during investigation of an abnormal screening mammogram. Breast Cancer Res Treat. 2002;76(3):245–254. 12462385 10.1023/a:1020820103126

[12] Lipkus IM, Halabi S, Strigo TS, et al. The impact of abnormal mammograms on psychosocial outcomes and subsequent screening. Psychooncology. 2000;9(5):402–410. 11038478 10.1002/1099-1611(200009/10)9:5<402::aid-pon475>3.0.co;2-u

[13] Brewer NT, Salz T, Lillie SE Systematic review: the long-term effects of false-positive mammograms. Ann Intern Med. 2007;146(7):502–510. 17404352 10.7326/0003-4819-146-7-200704030-00006

[14] Schou Bredal I, Karesen R, Skaane P, et al. Recall mammography and psychological distress. Eur J Cancer (Oxford, England: 1990). 2013;49(4):805–811. 10.1016/j.ejca.2012.09.001 23021930

[15] Hafslund B, Espehaug B, Nortvedt MW Effects of false-positive results in a breast screening program on anxiety, depression and health-related quality of life. Cancer Nurs. 2012;35(5):E26–E34. 22067696 10.1097/NCC.0b013e3182341ddb

[16] Brodersen J, Siersma VD Long-term psychosocial consequences of false-positive screening mammography. Ann Fam Med. 2013;11(2):106–115. 23508596 10.1370/afm.1466 PMC3601385

[17] DeFrank JT, Barclay C, Sheridan S, et al. The psychological harms of screening: the evidence we have versus the evidence we need. J Gen Intern Med. 2015;30(2):242–248. 25150033 10.1007/s11606-014-2996-5 PMC4314481

[18] Brodersen J, McKenna SP, Doward LC, et al. Measuring the psychosocial consequences of screening. Health Qual Life Outcomes. 2007;5:3. 17210071 10.1186/1477-7525-5-3 PMC1770907

[19] Brodersen J, Thorsen H, Cockburn J The adequacy of measurement of short and long-term consequences of false-positive screening mammography. J Med Screen. 2004;11(1):39–44. 15006113 10.1177/096914130301100109

[20] Roman M, Skaane P, Hofvind S The cumulative risk of false-positive screening results across screening centres in the Norwegian Breast Cancer Screening Program. Eur J Radiol. 2014;83(9):1639–1644. 24972452 10.1016/j.ejrad.2014.05.038

[21] Brodersen J, Thorsen H, Kreiner S Validation of a condition-specific measure for women having an abnormal screening mammography. ValueHealth. 2007;10(4):294–304. 10.1111/j.1524-4733.2007.00184.x 17645684

[22] Brodersen J, Thorsen H Consequences of Screening in Breast Cancer (COS-BC): development of a questionnaire. Scand J Prim Health Care. 2008;26(4):251–256. 19034808 10.1080/02813430802542508 PMC3406644

[23] Brodersen J, Thorsen H, Kreiner S Consequences of screening in lung cancer: development and dimensionality of a questionnaire. Value Health. 2010;13(5):601–612. 20345552 10.1111/j.1524-4733.2010.00697.x

[24] Swaine-Verdier A, Doward LC, Hagell P, et al. Adapting quality of life instruments. Value Health. 2004;7:27–30. 10.1111/j.1524-4733.2004.7s107.x 15367241

[25] Collins D Pretesting survey instruments: an overview of cognitive methods. Qual Life Res. 2003;12(3):229–238. 12769135 10.1023/a:1023254226592

[26] Andersen E A goodness of fit test for the rasch model. Psychometrika. 1973;38(1):123–140.

[27] Kreiner S Introduction to DIGRAM. Copenhagen: Department of Biostatistics Research, University of Copenhagen; 2003. Report No.: 03/10.

[28] Bolejko A, Hagell P, Wann-Hansson C, et al. Prevalence, long-term development, and predictors of psychosocial consequences of false-positive mammography among women attending population-based screening. Cancer Epidemiol Biomarkers Prev. 2015;24(9):1388–1397. 26311562 10.1158/1055-9965.EPI-15-0060

[29] Brett J, Bankhead C, Henderson B, Watson E, et al. The psychological impact of mammographic screening. A systematic review. Psychooncology. 2005;14(11):917–938. 15786514 10.1002/pon.904

[30] Lindberg L, Svendsen M, Dømgaard M, et al. Better safe than sorry: a long-term perspective on experiences with a false-positive screening mammography in Denmark. Health Risk Soc. 2013;15(8):699.

[31] Bond M, Garside R, Hyde C A crisis of visibility: the psychological consequences of false-positive screening mammograms, an interview study. Br J Health Psychol. 2015;20(4):792–806. 25944747 10.1111/bjhp.12142

[32] Solbjor M, Forsmo S, Skolbekken JA, et al. Experiences of recall after mammography screening–a qualitative study. Health Care Women Int. 2011;32(11):1009–1027. 21978146 10.1080/07399332.2011.565530

